# The Role of Early and Delayed Surgery in Return to Sport After Anterior Shoulder Dislocation—A Scoping Review

**DOI:** 10.3390/jcm14197045

**Published:** 2025-10-05

**Authors:** Martin Ingvardsen Vemmelund, Sten Rasmussen

**Affiliations:** Department of Clinical Medicine, Aalborg University, 9260 Gistrup, Denmark; sten@dcm.aau.dk

**Keywords:** anterior shoulder dislocation, athletes, surgical intervention, conservative treatment

## Abstract

**Background**: Anterior shoulder dislocations are common in athletes, particularly in contact sports. Surgical stabilization reduces recurrence, but the optimal timing—early versus delayed—remains uncertain, especially for in-season athletes. **Methods**: A systematic search of PubMed, Embase, and Cochrane (2013–2023) yielded 945 articles; 15 met the inclusion criteria. Data were charted on procedure type, outcomes, follow-up, patient group, and timing of surgery. Search terms, e.g., ‘shoulder’, ‘athlete’, ‘anterior’ and ‘shoulder dislocation’, were used in a broad search protocol casting a wide net to maximize the likelihood of capturing all available data. **Results**: Surgery was superior to conservative care in lowering recurrence and enabling return-to-play, with arthroscopic and combined procedures most effective in high-contact sports. Conservative management carried higher instability risk. Evidence directly comparing early versus delayed surgery was scarce, and therefore inconclusive. **Conclusions**: Surgical stabilization remains the treatment with better outcomes compared to conservative treatment for young athletes. Still, athletes opt to delay surgery until postseason, with the impact of delaying surgery being unclear. Further research is needed to evaluate early versus delayed surgery regarding recurrence, joint damage, and return to sport.

## 1. Introduction

Shoulder dislocations in young athletes pose a significant challenge, occurring more frequently than in the general population and leading to considerable morbidity and time away from sports [1,2,3]. Notably, anterior glenohumeral dislocations are most prevalent, especially in contact and collision sports, with 74.3% of all occurrences in males, placing young male athletes at a high risk [4,5]. Anterior shoulder dislocations can damage multiple structures in the glenohumeral joint. Common anatomical lesions include Bankart lesions, glenoid bone loss or fractures of the anterior glenoid rim, Hill–Sachs lesions, injuries to the capsule and glenohumeral ligaments, the long head of the biceps tendon and its insertion, and articular cartilage damage [6]. The impact of an in-season anterior glenohumeral dislocation extends beyond the individual athlete, affecting the entire team. Despite the prevalence of these injuries, there is currently no consensus on the optimal treatment for in-season anterior glenohumeral dislocations; initial treatment may include immobilization, rehabilitation, and return to activity or early shoulder stabilization [7]. Non-operative approaches, including immobilization followed by shoulder stabilizing exercises, can facilitate a swift return to sport within 7–21 days. However, one study suggests this expedited return is associated with a 92% risk of recurrent dislocations [7]. Consensus on nonoperative management after anterior shoulder dislocation is lacking, with protocols differing in immobilization position, duration, and rehabilitation. Reported strategies range from no immobilization to sling use for 1–6 weeks, with mixed outcomes—recurrence rates as high as 100% in adolescents after 6 weeks of sling use, versus high return-to-sport rates without immobilization in competitive athletes. While immobilization duration remains debated, most rapid return-to-play regimens advocate brief sling use (3–10 days) followed by phased rehabilitation, including cryotherapy, progressive strengthening of the rotator cuff and periscapular muscles, and sport-specific drills. Return-to-play is typically achieved within 3 weeks, though this varies individually [8]. The recurrence rates after surgery are significantly lower, ranging from 2 to 14.3% [7]. Despite the potential benefits of surgery in reducing recurrence, the lengthy recovery period often deters athletes from pursuing operative treatment while in-season, as it would result in substantial downtime and missed games [9]. This dilemma underscores the need for a nuanced understanding of the outcomes associated with athletes playing with anterior glenohumeral instability and delaying stabilizing surgery until the subsequent off-season, compared to those opting for acute stabilizing surgery.

The question is, what is the overall effect of early surgery? Could there be a benefit of early surgery compared to delayed surgery after anterior shoulder dislocation? A 2006 study evaluating 118 Bristow–Latarjet procedures for recurrent anterior shoulder dislocations reported that, at 15-year follow-up, most patients with preoperative arthropathy either showed no progression or even demonstrated improvement in their condition [10]. This scoping review aimed to evaluate the overall effect of early surgery for anterior shoulder dislocation in young active athletes. It is intuitive to assume that shoulder injuries affect athletes differently depending on the specific physical demands of their sport. For example, the stability and mobility requirements in soccer differ from those in handball. Nevertheless, in the present review, contact sports are considered as a single overarching category to allow for a broader synthesis of the available evidence.

## 2. Materials and Methods

This topic has not been comprehensively mapped, and the available literature is fragmented across different sports, treatment strategies, and follow-up designs. Unlike a systematic review, which is best suited to answer a narrowly defined clinical question by synthesizing comparable studies, the aim here was to explore the full extent and nature of existing evidence. Because our objective was to identify the breadth of available research, clarify key concepts, highlight patterns, and expose knowledge gaps—rather than test a focused hypothesis—a scoping review represented the most appropriate methodological choice. This approach enabled us to systematically chart diverse study designs and outcome measures, providing an overview that can inform the development of future high-quality research, including randomized controlled trials and meta-analyses.

A systematic search of the literature investigating the consequences of delayed versus acute stabilizing surgery was conducted across PubMed, Embase, and the Cochrane Library for the period 2013–2023. The most recent search was performed on 29 October 2024. Search terms included combinations of “shoulder dislocation,” “anterior,” “athlete,” “conservative treatment,” and “surgery.” Terms were applied as both free-text and controlled vocabulary (MeSH in PubMed and Emtree in Embase). The complete search strategy is provided in Appendix A—Systematic Literature Search. The search protocol was developed with the rationale of casting a wide net to maximize the likelihood of capturing all the available data, while limiting it to the last decade to reflect advances in surgical and conservative management techniques for athletes.

The initial search retrieved 945 records (27 from Cochrane Library, 506 from Embase, and 412 from PubMed).

**Figure 1:**
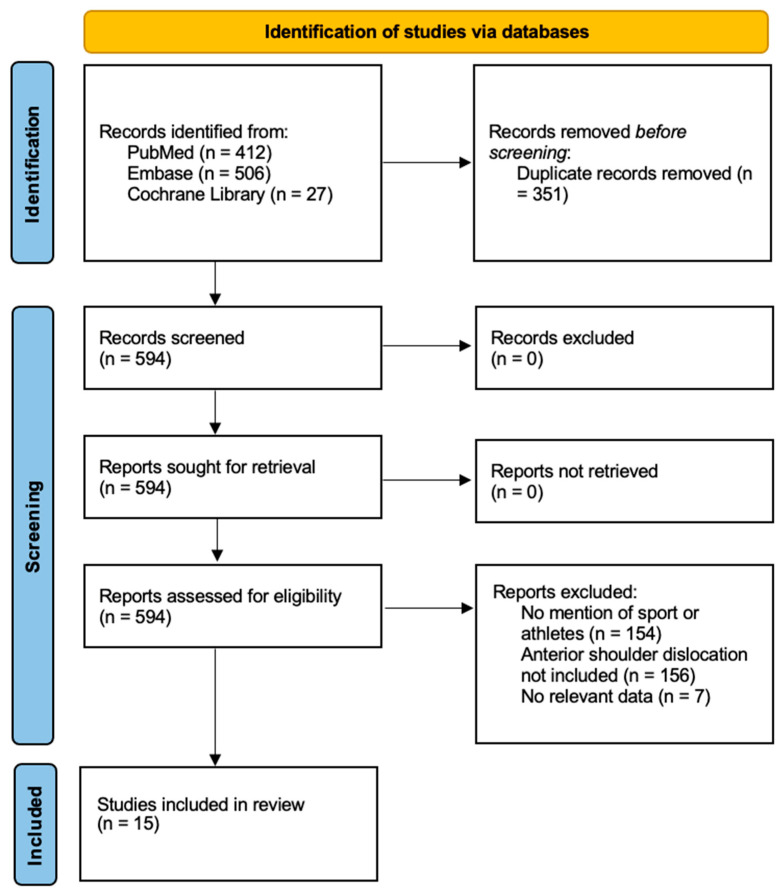
Flow chart that depicts the screening and exclusion process for this study.

Screening was conducted by one reviewer. A total of 351 were duplicates, 154 did not mention any kind of athletes or sports activities, and 156 did not include patients with anterior dislocations of the shoulder. Furthermore, seven studies were excluded because they did not contribute data relevant to evaluating the timing and outcomes of surgical stabilization after anterior shoulder dislocation in athletes. Procedural descriptions, meeting supplements, and study protocols lacked original outcome data. Case reports and cadaveric studies provided anecdotal or biomechanical insights but not clinical outcomes applicable to sports populations. Similarly, studies on the acromioclavicular (AC) joint and rehabilitation protocols for physiotherapists addressed conditions or interventions outside the scope of anterior glenohumeral dislocation management. As the objective of this scoping review was to map the breadth of clinical evidence concerning early versus delayed surgery in athletes, only studies with relevant populations, interventions, and outcomes were included. This leaves 15 articles. A flow diagram (Figure 1) illustrates this process.

Data from included studies were extracted using a standardized charting form. Data charting was performed by one reviewer. The extracted variables included study design, patient group, number of patients, type of sport, surgical procedure, time from injury to surgery, outcome measures (recurrence, return-to-sport, functional scores), and follow-up duration.

The main variables of interest were as follows:

1. Timing of surgery (early vs. delayed/postseason).
2. Type of intervention (arthroscopic Bankart, Latarjet, Bristow, conservative treatment, or combined procedures).
3. Scoring methods (recurrence rate, shoulder stability, functional outcome scores, or return-to-sport).
4. Population characteristics (age, sex, level of athletic competition, and sport type).
5. Follow-up duration and study setting.

Given the scoping review framework, no formal risk-of-bias tool was applied.

The charted data were synthesized descriptively. Key outcomes such as recurrence rates, return-to-sport timelines, and functional measures were summarized in narrative form, with patterns and gaps highlighted. Quantitative pooling was not attempted due to heterogeneity in study design, populations, and outcome reporting.

The 15 articles were reviewed regarding relevant information that could help showcase the role of early and late surgery in return to sports after anterior shoulder dislocation. The review of these articles focused on procedure, scoring method, time to follow-up, patient group, number of patients, and time from injury to surgery. 

This review follows the PRISMA-ScR guidelines but was not registered.

## 3. Results

### 3.1. Characteristics of Included Studies

The 15 included studies spanned diverse populations, ranging from recreational to elite athletes, some also including non-athletes, and investigated both surgical and nonoperative interventions. The key variables extracted included type of surgical procedure, intervention/procedure, outcome measures (recurrence rate, functional scores, return-to-sport), time to follow-up, sample size, and, where available, time from injury to surgery. A summary of study characteristics is provided in Table 1.

### 3.2. Critical Appraisal Within Sources of Evidence

No formal critical appraisal of methodological quality was conducted, in line with scoping review methodology, as the purpose was to map the extent and nature of available evidence rather than evaluate study validity. Nevertheless, methodological limitations noted by the primary studies (e.g., small cohorts, retrospective design) are considered in the synthesis.

### 3.3. Results of Individual Sources of Evidence

This review included a range of studies that investigated various surgical and nonoperative interventions for anterior shoulder dislocation, with a focus on athletic populations. The 15 articles mainly evaluated recurrence rates, functional outcomes, and the ability to return to sports after different treatment methods [7,8,11,12,13,14,15,16,17,18,19,20,21,22,23]. However, none investigated the timing of surgery, and only two studies mention the knowledge gap of the timing of surgical intervention [8,23].

#### 3.3.1. Timing of Surgery

There are currently no articles investigating the timing of surgery after anterior shoulder dislocation, and only refs. [8,19] have addressed the knowledge gap. The effect of delaying surgery is particularly relevant for athletes who may choose conservative treatment over surgery during in-season injuries to prevent prolonged time away from competition. Future studies should focus on understanding whether delaying surgery impacts outcomes such as recurrence, return-to-sport time, and overall shoulder stability in athletic populations.

#### 3.3.2. Surgical Interventions

Several studies supported the efficacy of arthroscopic and open surgical procedures for reducing recurrence and improving return-to-play outcomes in athletes with anterior shoulder dislocation [7,8,11,12,13,14,16,18,19,20,21,22]. Arthroscopic Bankart repair showed superiority over conservative treatment, especially regarding recurrence prevention and return to play, as seen in studies involving first-time traumatic anterior dislocations in young athletes [13]. Other studies (e.g., [14]) demonstrated that arthroscopic Bankart repair improved functional outcomes compared to immobilization alone, reinforcing surgical intervention as a preferable option in high-risk athletic populations. Any non-surgical interventions post-surgery, such as immobilization or exercise, have not been mentioned.

#### 3.3.3. Combined Procedures

Combined arthroscopic Bankart repair and coracoid process transfer to the anterior glenoid was proven effective in preventing recurrent dislocation in rugby players with no recurrent dislocations at a mean follow-up of 30.5 months. A total of 10 percent of the shoulders treated with this procedure reported limitations in specific sports-related movements [16]. In total, 23 patients with recurrent instability and Hill–Sachs lesions with mild glenoid bone loss were treated with the modified Latarjet procedure. No patients who underwent the procedure had recurrent dislocations; a significant portion of the patients had complications: three deep infections, two graft fractures, two painful hardware, one broken screw with graft malunion, and one radiographic graft nonunion [23].

#### 3.3.4. Outcomes in Diverse Athletic Populations

Outcomes varied based on the type of sport and level of competition. For instance, a treatment study indicated that open Bankart repairs were effective across various sports. The study included 127 patients; 107 participated in sports (overhead *n* = 29, contact *n* = 43, noncontact non-overhead *n* = 35). At a mean follow-up of 17.1 years, there was one recurrent dislocation (0.8%) in a woman with Ehlers–Danlos syndrome, and one recurrent subluxation (0.8%) in a woman with bipolar and seizure disorders [18]. A cohort study compared 57 competitive athletes to 49 recreational athletes, all under 30. Both groups were treated with an open Latarjet procedure. At a mean follow-up of 46 months, there was recurrence of shoulder luxation in two competitive athletes (3.5%) and one recreational athlete (2%). The persistent apprehension test result was positive in seven competitive athletes (11.5%) and in five recreational athletes (10%). The net improvement when comparing Rowe scores pre- and post-surgery was significantly higher in competitive athletes (27.9 ± 21.7) compared with recreational athletes (14.5 ± 24.4). In regard to return to sport, all 57 competitive athletes (100%) and 34 recreational athletes (69.4%) resumed their previous sports practice at the same level or higher than before their injury, suggesting competitive athletes benefit more from the open Latarjet procedure than recreational athletes [21].

Two systematic reviews [7,17] found that overhead athletes were more at risk of recurrence compared to other athletes. Chiddarwar et al. also found that athletes’ return to activity was longer than non-athletes’ after arthroscopic shoulder stabilization and exercise-based intervention (EBI). Non-athletes returned to activity 3–4 months post-surgery, while one study found that athletes returned to activity on average 8.4 months post-surgery [17].

#### 3.3.5. Nonoperative vs. Operative Management

Several articles consistently indicate that nonoperative management resulted in higher recurrent instability rates compared to surgical interventions [7,8,11,12,13,14,16,18,19,20,21,22]. For instance, a systematic review [7] investigated primary anterior shoulder dislocations and found that recurrence rates after conservative treatment for 10–13-year-olds were 21.4–23%, for 30–40-year-olds this number was 17%, and for patients under 30 this number was 47%. This suggests that operative management should be prioritized for young, athletic patients, as they present a higher risk of recurrent dislocations compared to older individuals. This review also found that 88.6% of 15- to 25-year-old athletes undergoing nonoperative management returned to sport, and 71.4% experienced recurrent dislocations. Of those undergoing surgical intervention, 82.5% to 93.3% returned to sport with return to pre-injury performance rates of 69% to 90%. While athletes treated conservatively could return to sport in as little as 21 days, athletes who are in-season and undergo operative management will not be able to return to sport during the same season due to the necessary rehabilitation following surgery [7]. 

#### 3.3.6. Combined Surgical and Exercise-Based Interventions (EBI)

A systematic review and meta-analysis, which included 67 studies, investigated recurrence, return to activity, self-reported measures, and physical measures (e.g., range of motion and strength). The meta-analysis found that patients who underwent surgery followed by EBI were 2.03 times less likely to experience recurrence, and 1.81 times more likely to return to activity than patients who had EBI alone. Self-reported measures were found either insignificant or in favor of surgery followed by EBI. For strength and range of motion, the results were mostly in favor of EBI alone, with many of the results being insignificant. The qualitative analysis showed better outcomes in terms of recurrence when the athletes did not return to sport at the pre-injury level within the same season, indicating that EBI supported return to sport after surgery. Regarding self-reported measures, the studies analyzed often lacked a control group but reported improved outcomes when compared to preoperative measures [17]. This suggests that rehabilitation protocols may enhance the advantages of surgical stabilization.

### 3.4. Synthesis of Results

Overall, surgical stabilization demonstrated superiority over conservative approaches in young athletes, particularly regarding recurrence and return to play. However, evidence concerning the timing of surgery—early versus delayed—is extremely limited and inconclusive. The synthesis underscores a significant knowledge gap with high clinical relevance, given that many athletes delay surgery until postseason to remain active during competition.

## 4. Discussion

### 4.1. Summary of Evidence

This scoping review was unable to definitively answer the research question regarding the benefits of early versus delayed surgery for anterior shoulder dislocations in young athletes. The review identified no data on the timing of surgery. Nonetheless, the available evidence consistently indicates that surgical stabilization provides superior outcomes compared with conservative management, particularly with respect to reducing recurrence rates and enabling a return to pre-injury performance levels in young adults involved with contact and overhead sports. For 30–40-year-old patients, recurrence after conservative treatment was found to be 17%, while patients below the age of 30 had a recurrence rate of 47% following conservative treatment [7,9,11,12,13,14,16,18,19,20,21,22].

Delaying surgery may allow athletes to complete a competitive season, but this approach potentially increases the risk of recurrent instability, especially for athletes involved with contact and overhead sports [7]. With every dislocation, there is a risk of damage to ligaments, capsule changes, cuff tears, vascular injury, neural injury, cartilage, and osseous lesions, which can further compromise long-term shoulder function [6,24]. With stabilizing surgery, the risk of further arthropathy is minimized, and the chance of improvement is higher than if treated conservatively [10]. Evidence from high-contact sports, such as rugby, suggests that recurrent instability may necessitate more complex procedures, including Latarjet or Bristow operations [16,23].

### 4.2. Limitations of the Review

Several limitations of this review must be acknowledged. First, the number of eligible studies was limited, and none investigated surgical timing, restricting our ability to provide definitive conclusions. Second, heterogeneity in study design, outcome measures, and follow-up times hindered direct comparisons across studies. Third, the review was limited to articles published in the past decade, which may have excluded relevant earlier literature. Fourth, only one author reviewed the articles included in this study, which could lead to bias. Finally, no formal critical appraisal of study quality was performed, consistent with scoping review methodology, but this may limit the interpretation of the strength of evidence.

### 4.3. Implications and Future Directions

The most pressing evidence gap concerns the effect of surgical timing—early versus delayed—on long-term effects in young athletes competing in contact or overhead sports. The literature indicates that this patient group benefits from stabilizing surgery more than patients above 30 and below 13 years of age. The young athletes have a higher risk of recurrence if treated conservatively, which can lead to further damage to the joint. Whether this has any effects on the long-term outcomes for the athlete is unknown. Addressing this question should be prioritized as it is an important aspect when opting for either surgery or conservative treatment, especially during the competitive season. Although randomized controlled trials would provide the highest level of evidence, they are difficult to implement in this population due to ethical concerns, small eligible cohorts, and athletes’ reluctance to accept randomization that may jeopardize their careers. As such, large prospective multicenter cohort studies, pragmatic trials, and registry-based research represent more feasible approaches and could still generate high-quality, generalizable data. Also, retrospective studies, where the patients are divided into groups depending on the timing of surgery after anterior shoulder dislocation, would be useful. This would enable the study to investigate the long-term effects sooner than a prospective study. Integrating sport-specific stratification into future studies may also clarify whether the impact of surgical timing varies across different contact sports. Collectively, such evidence would form a more practical foundation for developing evidence-based guidelines and supporting individualized decision-making in clinical practice.

## 5. Conclusions

The management of anterior shoulder dislocations in athletes during the competitive season presents a complex challenge that lacks a clear, evidence-based solution. Current evidence indicates that surgical intervention is the most effective method for preventing recurrence and facilitating a return to sports in young athletes; however, the optimal timing for this intervention remains uncertain. Early surgery reduces the risk of recurrent instability but requires a significant amount of downtime during the season. On the other hand, delaying surgery may enable athletes to keep competing, but this comes with the risk of additional dislocations and ongoing joint damage.

This scoping review highlights a significant gap in the literature regarding the outcomes of delaying surgical intervention. It is essential to address this knowledge gap to help both clinicians and athletes make informed, personalized treatment decisions. Comprehensive studies are needed to examine the long-term impacts of delayed surgery on recurrence rates, joint integrity, and return-to-sport outcomes. This research will better inform clinical practice and optimize recovery strategies for athletes with anterior shoulder dislocations. Ultimately, clear evidence on the consequences of playing with an unstable shoulder will provide a foundation for establishing definitive treatment guidelines. This will aid athletes and medical professionals in navigating this complex decision-making process.

## Figures and Tables

**Table 1:** The 15 articles for review compared procedure, score-method, outcomes, follow-up time, number of patients, patient group, time from injury to surgery, and conclusion. ROM = Range of motion. CMS = Constant-Murley Score. DASH = The Disability of the arm, shoulder, and Hand questionnaire. VAS = Visual Analogue Scale. ASES = American Shoulder and Elbow Surgeons scale. ISIS = Instability Severity Index Score. OSIS = Oxford Shoulder Instability Score. WOSI = Western Ontario Shoulder Instability Index. SANE score = Single Assessment Numeric score.

Article	Procedure	Score-Method/Outcomes	Follow-Up Time	Number of Patients	Patient Group	Time from Injury to Surgery	Conclusion
Kraeutler, et al. [3]	Nonoperative management Open Bankart Repair Arthroscopic Bankart Repair	Recurrence rate	Systematic review, Unknown	Systematic review, Unknown	Anterior shoulder dislocations No specific sports mentioned	Unknown	“Operative management should be considered as a treatment option for young and athletic patients presenting with a first-time anterior GHJ dislocation” “Nonoperative man- agreement may be indicated in patients older than 30 years, who are at a lower risk of recurrence.”
Watson, et al. [8]	Primary conservative treatment Secondary surgical intervention	Recurrence rate Return to play	Unknown	Unknown	Athletes Both collision, limited contact, and no contact	“There is a limited amount of evidence available to draw a conclusion on timing of surgery and whether return to play during the season affects outcomes.” Unknown	“While the majority of cases eventually result in surgical stabilization, the timing to surgery (…) remain controversial”
Clesham, et al. [11]	Arthroscopic stabilization	WOSI Return to play Recurrence rate	Mean: 5.04 years	*n* = 90 Follow-up: 61	Athletes Overhead collisions sports compared to other sports	Unknown	“Athletes with recurrent glenohumeral instability involved in overhead collision sports can be treated effectively with arthroscopic stabilization”
Tasaki, et al. [12]	Combined arthroscopic Bankart Repair and Open Bristow Procedure	Rowe Score Recurrence rate Glenoid diameter and area	Mean: 3.4 years	*n* = 141 149 shoulders	Collision athletes	Unknown	“(…) a combined arthroscopic Bankart repair and open Bristow procedure is effective in treating traumatic anterior shoulder instability in collision sports athletes with or without osseous glenoid lesions”
Hu, et al. [13]	Arthroscopic Bankart repair versus conservative treatment	Recurrence rate Return to play Subsequent instability surgery Constant-Murley score DASH score Rowe score ASES score WOSI score	Mean: 4.13 years	12 trials 786 patients	First-time traumatic anterior shoulder dislocation in young population (Mean age: 21.7 years)	Unknown	“Arthroscopic Bankart repair showed superiority over conservative management in terms of recurrence, return to play, and subsequent instability surgery” “Outcomes regarding the functional scores did not reach a significant difference”
Pougés, et al. [14]	Arthroscopic Bankart repair versus Immobilization	WOSI The Walch-Duplay score Recurrence rate	2 years	40 patients (20 surgery and 20 immobilization)	First-time anterior shoulder dislocation No specific sports mentioned Mean age: 21 82.5% men, 17.5% women	Unknown	“(…) arthroscopic Bankart procedure reduced the risk of secondary shoulder dislocation and improved functional outcome vs. nonoperative treatment after er 2-year follow-up”
El-Sofy, et al. [15]	Latarjet procedure	Griffith Index Bigliana classification Gleniod Boneloss	Unknown	Unknown	Anterior shoulder dislocation, both first time and recurrence. No specific sports mentioned.	Unknown	“The Latarjet procedure remains the gold standard technique for treating anterior glenoid bone loss, and its success is evidenced by its low long-term failure rate. Several studies suggest excellent outcomes for both traditional and congruent arc techniques; however, there have been no clinical studies comparing the two methods”
Tasaki, et al. [16]	Combined Arthroscopic Bankart Repair and Bristow procedure	Ability to perform sport-specific movements effectively ROWE score	Mean: 2.54 years	38 individuals 40 shoulders	Rugby Players Mean age: 21 years	Unknown	“This combined surgical procedure is effective in preventing recurrent shoulder dislocation in rugby players; however, some players (10%) complained of insufficiency in the quality of their play when they were tackling or performing other specific movements”
Chiddarwar, et al. [17]	Combined surgical and exercise-based interventions No specifics mentioned	Recurrence Return to activity ASES CMS DASH ROWE score VAS WOSI Shoulder Muscle Strength Shoulder ROM	Mean: 0.9 years	*n* = 3943	Primary traumatic anterior shoulder dislocations No specific sports mentioned Mean age: 26.71 years 56% men, 44% women	Unknown	“Surgery combined with exercise-based interventions is more effective in reducing the risk of recurrence and possibly increasing return to activity than exercise-based interventions alone after traumatic anterior shoulder dislocation”
Do, et al. [18]	Conservative treatment	Beighton score	2 years	*n* = 61	Anterior shoulder dislocations No specific sports mentioned Mean age: 28 years 85% men, 15% women	Unknown/No surgery	“(…) instability occurred in 75,4% of patients”
Hasebroock, et al. [19]	Conservative versus surgery	ISIS	Unknown	Unknown	Primary anterior shoulder dislocations No specific sports mentioned All ages	“Timely management of anterior shoulder dislocations is absolutely essential for optimal patient outcomes, as there is elevated risk of unstable reduction if the shoulder is left untreated for over 24 h from initial injury” Unknown	“Additional randomized controlled trials are necessary to further explore optimal long-term management. Cur- rently, recommendations for unrestricted return to play remain broad and generalized, as opposed to a specific timeline.”
Neviaser, et al. [20]	Open Bankart repair	ROM Arthritis Osteolysis ASES Rowe score WOSI	Mean: 17.1 years	*n* = 127	Recurrent, traumatic anterior shoulder dislocation Including contact sports, overhead sports, noncontact sports, and nonoverhead sports	Unknown	“(…) the open Bankart remains the standard by which other techniques can be measured for the treatment of recurrent, traumatic anterior dislocation of the shoulder”
Baveral, et al. [21]	Open Latarjet procedure	ISIS Rowe score OSIS WOSI VAS Subjective Shoulder Value	Mean: 3.83 years	*n* = 106 110 shoulders	16< age < 30 Competitive Athletes and Recreational Athletes Both contact/collision, limited contact, and noncontact sports. No specific sports mentioned	Unknown	“(…) the open latarjet procedure rendered good outcomes for competitive and recreational athletes, with equivalent redislocation rates in both groups (5%). The clinical scores and rates of return to sports were, however, significantly better for competitive athletes, regardless of the type of sport and level of competition.”
Donohue, et al. [22]	Nonoperative (Immobilization, Physical therapy, and Brace wear) Surgery (Arthroscopic repair, Open repair, and Open osseous augmentation)	Return to play	Unknown	Unknown	Athletes involved in various sports at all levels.	Unknown	“Arthroscopic and open Bankart repair are both reliable treatment options with a trend towards decreased recurrence using open Bankart repair for contact and collision athletes.” “Open osseous augmentation procedures should be used for athletes with greater than 20% to 25% glenoid bone loss.”
Yang, et al. [23]	Modified Latarjet, 61% had previous open or arthroscopic stabilization procedures	Glenoid bone loss ISIS WOSI SANE score Recurrence rate Return to sport	Mean: 3.5 years	*n* = 23	Recurrent, anterior shoulder instability, engaging Hill–Sachs, and less than 25% anterior glenoid bone loss.	Unknown	“The modified Latarjet is a reasonable choice for the treatment of an engaging Hill–Sachs lesion with concomitant mild anterior glenoid bone loss in this very demanding group of patients. However, the complication rate is significant.”

## Data Availability

The original contributions presented in this study are included in the article. Further inquiries can be directed to the corresponding author(s).

## Appendix A

Systematic Literature Search Protocol:

Search term blocks:

1. Shoulder dislocation	MeSH: Shoulder Dislocation Bankart Lesions Joint Instability (**AND** shoulder OR glenohum* OR gleno-hum* OR labrum)ti,ab Athletic Injuries (**AND** shoulder OR glenohum* OR gleno-hum* OR labrum)ti,ab Emtree: Shoulder Dislocation Bankart Lesions Joint Instability/de (**AND** shoulder OR glenohum* OR gleno-hum* OR labrum)ti,ab Sport injury (**AND** shoulder OR glenohum* OR gleno-hum* OR labrum)ti,ab Text: (shoulder OR glenohum* OR gleno-hum* OR labrum) **AND** ([15]nstability* OR dislocation* OR subluxation* OR luxation*) OR “Bankart lesion*” OR “Bankart fracture*”
2. Anterior	MeSH: - Emtree: - Text: Anterior
3. Surgery/Conservative treatment	MeSH: Shoulder Joint/surgery Orthopedics Surgical Procedures, Operative Conservative Treatment Emtree: Orthopedics Orthopedic surgery Conservative treatment Text: operat* OR surger* OR surgic* OR “Bankart repair*” OR “Bankart procedure*” OR “Latarjet-Bristow*” OR conservative OR immobilis* OR immobiliz* OR “collar and cuff” OR “collarandcuff” OR non-surg* OR nonsurg* OR non-operat* OR nonoperat*
4. Athletes	MeSH: Athletes Sports Athletic Injuries Emtree: Athlete Sport Sport injury Text: athlete* OR sport* OR competition* OR play OR collision*
5. Outcome	MeSH: Treatment outcome Return to sport Emtree: Treatment outcome Return to play Return to sport Text: “return to play*” OR “return to sport*” OR “return to competition*” OR outcome OR resumption* OR “treatment efficacy”

Database search history:

PubMed:

**Search Number**	**Search Details**	**Results**
23	(“Shoulder Dislocation”[MeSH Terms] OR “Bankart Lesions”[MeSH Terms] OR ((“Joint Instability”[MeSH Terms] OR “Athletic Injuries”[MeSH Terms]) AND (“shoulder”[Title/Abstract] OR “glenohum*”[Title/Abstract] OR “gleno hum*”[Title/Abstract] OR “labrum”[Title/Abstract])) OR ((“shoulder”[Title/Abstract] OR “glenohum*”[Title/Abstract] OR “gleno hum*”[Title/Abstract] OR “labrum”[Title/Abstract]) AND (“[17]nstability*”[Title/Abstract] OR “dislocation*”[Title/Abstract] OR “subluxation*”[Title/Abstract] OR “luxation*”[Title/Abstract])) OR (“bankart lesion*”[Title/Abstract] OR “bankart fracture*”[Title/Abstract])) AND “anterior”[Title/Abstract] AND (“shoulder joint/surgery”[MeSH Terms] OR “Orthopedics”[MeSH Terms] OR “surgical procedures, operative”[MeSH Terms] OR “Conservative Treatment”[MeSH Terms] OR (“operat*”[Title/Abstract] OR “surger*”[Title/Abstract] OR “surgic*”[Title/Abstract] OR “bankart repair*”[Title/Abstract] OR “bankart procedure*”[Title/Abstract] OR “latarjet bristow*”[Title/Abstract] OR “conservative”[Title/Abstract] OR “immobilis*”[Title/Abstract] OR “immobiliz*”[Title/Abstract] OR “collar and cuff”[Title/Abstract] OR “collarandcuff”[Title/Abstract] OR “non surg*”[Title/Abstract] OR “nonsurg*”[Title/Abstract] OR “non operat*”[Title/Abstract] OR “nonoperat*”[Title/Abstract])) AND (“Athletes”[MeSH Terms] OR “Sports”[MeSH Terms] OR “Athletic Injuries”[MeSH Terms] OR (“athlete*”[Title/Abstract] OR “sport*”[Title/Abstract] OR “competition*”[Title/Abstract] OR “play”[Title/Abstract] OR “collision*”[Title/Abstract])) AND (“[17]nstabi”[Language] OR “[17]nstab”[Language]) AND 2013/01/01:2023/12/31[Date – Publication] AND (“Treatment Outcome”[MeSH Terms] OR “Return to Sport”[MeSH Terms] OR (“return to play*”[Title/Abstract] OR “return to sport*”[Title/Abstract] OR “return to competition*”[Title/Abstract] OR “outcome”[Title/Abstract] OR “resumption*”[Title/Abstract] OR “treatment efficacy”[Title/Abstract]))	412
22	“Treatment Outcome”[MeSH Terms] OR “Return to Sport”[MeSH Terms] OR “return to play*”[Title/Abstract] OR “return to sport*”[Title/Abstract] OR “return to competition*”[Title/Abstract] OR “outcome”[Title/Abstract] OR “resumption*”[Title/Abstract] OR “treatment efficacy”[Title/Abstract]	2,279,835
21	“return to play*”[Title/Abstract] OR “return to sport*”[Title/Abstract] OR “return to competition*”[Title/Abstract] OR “outcome”[Title/Abstract] OR “resumption*”[Title/Abstract] OR “treatment efficacy”[Title/Abstract]	1,316,819
20	“Treatment Outcome”[MeSH Terms] OR “Return to Sport”[MeSH Terms]	1,254,697
19	(“Shoulder Dislocation”[MeSH Terms] OR “Bankart Lesions”[MeSH Terms] OR ((“Joint Instability”[MeSH Terms] OR “Athletic Injuries”[MeSH Terms]) AND (“shoulder”[Title/Abstract] OR “glenohum*”[Title/Abstract] OR “gleno hum*”[Title/Abstract] OR “labrum”[Title/Abstract])) OR ((“shoulder”[Title/Abstract] OR “glenohum*”[Title/Abstract] OR “gleno hum*”[Title/Abstract] OR “labrum”[Title/Abstract]) AND (“[18]nstability*”[Title/Abstract] OR “dislocation*”[Title/Abstract] OR “subluxation*”[Title/Abstract] OR “luxation*”[Title/Abstract])) OR (“bankart lesion*”[Title/Abstract] OR “bankart fracture*”[Title/Abstract])) AND “anterior”[Title/Abstract] AND (“shoulder joint/surgery”[MeSH Terms] OR “Orthopedics”[MeSH Terms] OR “surgical procedures, operative”[MeSH Terms] OR “Conservative Treatment”[MeSH Terms] OR (“operat*”[Title/Abstract] OR “surger*”[Title/Abstract] OR “surgic*”[Title/Abstract] OR “bankart repair*”[Title/Abstract] OR “bankart procedure*”[Title/Abstract] OR “latarjet bristow*”[Title/Abstract] OR “conservative”[Title/Abstract] OR “immobilis*”[Title/Abstract] OR “immobiliz*”[Title/Abstract] OR “collar and cuff”[Title/Abstract] OR “collarandcuff”[Title/Abstract] OR “non surg*”[Title/Abstract] OR “nonsurg*”[Title/Abstract] OR “non operat*”[Title/Abstract] OR “nonoperat*”[Title/Abstract])) AND (“Athletes”[MeSH Terms] OR “Sports”[MeSH Terms] OR “Athletic Injuries”[MeSH Terms] OR (“athlete*”[Title/Abstract] OR “sport*”[Title/Abstract] OR “competition*”[Title/Abstract] OR “play”[Title/Abstract] OR “collision*”[Title/Abstract])) AND (“[18]nstabi”[Language] OR “[18]nstab”[Language]) AND 2013/01/01:2023/12/31[Date – Publication]	“Outcome” block left out 652
18	(“Shoulder Dislocation”[MeSH Terms] OR “Bankart Lesions”[MeSH Terms] OR ((“Joint Instability”[MeSH Terms] OR “Athletic Injuries”[MeSH Terms]) AND (“shoulder”[Title/Abstract] OR “glenohum*”[Title/Abstract] OR “gleno hum*”[Title/Abstract] OR “labrum”[Title/Abstract])) OR ((“shoulder”[Title/Abstract] OR “glenohum*”[Title/Abstract] OR “gleno hum*”[Title/Abstract] OR “labrum”[Title/Abstract]) AND (“[18]nstability*”[Title/Abstract] OR “dislocation*”[Title/Abstract] OR “subluxation*”[Title/Abstract] OR “luxation*”[Title/Abstract])) OR (“bankart lesion*”[Title/Abstract] OR “bankart fracture*”[Title/Abstract])) AND “anterior”[Title/Abstract] AND (“shoulder joint/surgery”[MeSH Terms] OR “Orthopedics”[MeSH Terms] OR “surgical procedures, operative”[MeSH Terms] OR “Conservative Treatment”[MeSH Terms] OR (“operat*”[Title/Abstract] OR “surger*”[Title/Abstract] OR “surgic*”[Title/Abstract] OR “bankart repair*”[Title/Abstract] OR “bankart procedure*”[Title/Abstract] OR “latarjet bristow*”[Title/Abstract] OR “conservative”[Title/Abstract] OR “immobilis*”[Title/Abstract] OR “immobiliz*”[Title/Abstract] OR “collar and cuff”[Title/Abstract] OR “collarandcuff”[Title/Abstract] OR “non surg*”[Title/Abstract] OR “nonsurg*”[Title/Abstract] OR “non operat*”[Title/Abstract] OR “nonoperat*”[Title/Abstract])) AND (“Athletes”[MeSH Terms] OR “Sports”[MeSH Terms] OR “Athletic Injuries”[MeSH Terms] OR (“athlete*”[Title/Abstract] OR “sport*”[Title/Abstract] OR “competition*”[Title/Abstract] OR “play”[Title/Abstract] OR “collision*”[Title/Abstract])) AND (“[19]nstabi”[Language] OR “[19]nstab”[Language])	1097
17	(“Shoulder Dislocation”[MeSH Terms] OR “Bankart Lesions”[MeSH Terms] OR ((“Joint Instability”[MeSH Terms] OR “Athletic Injuries”[MeSH Terms]) AND (“shoulder”[Title/Abstract] OR “glenohum*”[Title/Abstract] OR “gleno hum*”[Title/Abstract] OR “labrum”[Title/Abstract])) OR ((“shoulder”[Title/Abstract] OR “glenohum*”[Title/Abstract] OR “gleno hum*”[Title/Abstract] OR “labrum”[Title/Abstract]) AND (“[19]nstability*”[Title/Abstract] OR “dislocation*”[Title/Abstract] OR “subluxation*”[Title/Abstract] OR “luxation*”[Title/Abstract])) OR (“bankart lesion*”[Title/Abstract] OR “bankart fracture*”[Title/Abstract])) AND “anterior”[Title/Abstract] AND (“shoulder joint/surgery”[MeSH Terms] OR “Orthopedics”[MeSH Terms] OR “surgical procedures, operative”[MeSH Terms] OR “Conservative Treatment”[MeSH Terms] OR (“operat*”[Title/Abstract] OR “surger*”[Title/Abstract] OR “surgic*”[Title/Abstract] OR “bankart repair*”[Title/Abstract] OR “bankart procedure*”[Title/Abstract] OR “latarjet bristow*”[Title/Abstract] OR “conservative”[Title/Abstract] OR “immobilis*”[Title/Abstract] OR “immobiliz*”[Title/Abstract] OR “collar and cuff”[Title/Abstract] OR “collarandcuff”[Title/Abstract] OR “non surg*”[Title/Abstract] OR “nonsurg*”[Title/Abstract] OR “non operat*”[Title/Abstract] OR “nonoperat*”[Title/Abstract])) AND (“Athletes”[MeSH Terms] OR “Sports”[MeSH Terms] OR “Athletic Injuries”[MeSH Terms] OR (“athlete*”[Title/Abstract] OR “sport*”[Title/Abstract] OR “competition*”[Title/Abstract] OR “play”[Title/Abstract] OR “collision*”[Title/Abstract]))	1197
16	“Athletes”[MeSH Terms] OR “Sports”[MeSH Terms] OR “Athletic Injuries”[MeSH Terms] OR “athlete*”[Title/Abstract] OR “sport*”[Title/Abstract] OR “competition*”[Title/Abstract] OR “play”[Title/Abstract] OR “collision*”[Title/Abstract]	1,267,809
15	“athlete*”[Title/Abstract] OR “sport*”[Title/Abstract] OR “competition*”[Title/Abstract] OR “play”[Title/Abstract] OR “collision*”[Title/Abstract]	1,108,848
14	“Athletes”[MeSH Terms] OR “Sports”[MeSH Terms] OR “Athletic Injuries”[MeSH Terms]	240,19
13	(“Shoulder Dislocation”[MeSH Terms] OR “Bankart Lesions”[MeSH Terms] OR ((“Joint Instability”[MeSH Terms] OR “Athletic Injuries”[MeSH Terms]) AND (“shoulder”[Title/Abstract] OR “glenohum*”[Title/Abstract] OR “gleno hum*”[Title/Abstract] OR “labrum”[Title/Abstract])) OR ((“shoulder”[Title/Abstract] OR “glenohum*”[Title/Abstract] OR “gleno hum*”[Title/Abstract] OR “labrum”[Title/Abstract]) AND (“[19]nstability*”[Title/Abstract] OR “dislocation*”[Title/Abstract] OR “subluxation*”[Title/Abstract] OR “luxation*”[Title/Abstract])) OR (“bankart lesion*”[Title/Abstract] OR “bankart fracture*”[Title/Abstract])) AND “anterior”[Title/Abstract] AND (“shoulder joint/surgery”[MeSH Terms] OR “Orthopedics”[MeSH Terms] OR “surgical procedures, operative”[MeSH Terms] OR “Conservative Treatment”[MeSH Terms] OR (“operat*”[Title/Abstract] OR “surger*”[Title/Abstract] OR “surgic*”[Title/Abstract] OR “bankart repair*”[Title/Abstract] OR “bankart procedure*”[Title/Abstract] OR “latarjet bristow*”[Title/Abstract] OR “conservative”[Title/Abstract] OR “immobilis*”[Title/Abstract] OR “immobiliz*”[Title/Abstract] OR “collar and cuff”[Title/Abstract] OR “collarandcuff”[Title/Abstract] OR “non surg*”[Title/Abstract] OR “nonsurg*”[Title/Abstract] OR “non operat*”[Title/Abstract] OR “nonoperat*”[Title/Abstract]))	4193
12	“shoulder joint/surgery”[MeSH Terms] OR “Orthopedics”[MeSH Terms] OR “surgical procedures, operative”[MeSH Terms] OR “Conservative Treatment”[MeSH Terms] OR “operat*”[Title/Abstract] OR “surger*”[Title/Abstract] OR “surgic*”[Title/Abstract] OR “bankart repair*”[Title/Abstract] OR “bankart procedure*”[Title/Abstract] OR “latarjet bristow*”[Title/Abstract] OR “conservative”[Title/Abstract] OR “immobilis*”[Title/Abstract] OR “immobiliz*”[Title/Abstract] OR “collar and cuff”[Title/Abstract] OR “collarandcuff”[Title/Abstract] OR “non surg*”[Title/Abstract] OR “nonsurg*”[Title/Abstract] OR “non operat*”[Title/Abstract] OR “nonoperat*”[Title/Abstract]	5,593,339
11	“operat*”[Title/Abstract] OR “surger*”[Title/Abstract] OR “surgic*”[Title/Abstract] OR “bankart repair*”[Title/Abstract] OR “bankart procedure*”[Title/Abstract] OR “latarjet bristow*”[Title/Abstract] OR “conservative”[Title/Abstract] OR “immobilis*”[Title/Abstract] OR “immobiliz*”[Title/Abstract] OR “collar and cuff”[Title/Abstract] OR “collarandcuff”[Title/Abstract] OR “non surg*”[Title/Abstract] OR “nonsurg*”[Title/Abstract] OR “non operat*”[Title/Abstract] OR “nonoperat*”[Title/Abstract]	3,382,305
10	“shoulder joint/surgery”[MeSH Terms] OR “Orthopedics”[MeSH Terms] OR “surgical procedures, operative”[MeSH Terms] OR “Conservative Treatment”[MeSH Terms]	3,577,768
9	“anterior”[Title/Abstract]	429,328
8	“Shoulder Dislocation”[MeSH Terms] OR “Bankart Lesions”[MeSH Terms] OR ((“Joint Instability”[MeSH Terms] OR “Athletic Injuries”[MeSH Terms]) AND (“shoulder”[Title/Abstract] OR “glenohum*”[Title/Abstract] OR “gleno hum*”[Title/Abstract] OR “labrum”[Title/Abstract])) OR ((“shoulder”[Title/Abstract] OR “glenohum*”[Title/Abstract] OR “gleno hum*”[Title/Abstract] OR “labrum”[Title/Abstract]) AND (“[20]nstability*”[Title/Abstract] OR “dislocation*”[Title/Abstract] OR “subluxation*”[Title/Abstract] OR “luxation*”[Title/Abstract])) OR (“bankart lesion*”[Title/Abstract] OR “bankart fracture*”[Title/Abstract])	17,033
7	“bankart lesion*”[Title/Abstract] OR “bankart fracture*”[Title/Abstract]	828
6	(“shoulder”[Title/Abstract] OR “glenohum*”[Title/Abstract] OR “gleno hum*”[Title/Abstract] OR “labrum”[Title/Abstract]) AND (“[21]nstability*”[Title/Abstract] OR “dislocation*”[Title/Abstract] OR “subluxation*”[Title/Abstract] OR “luxation*”[Title/Abstract])	13,371
5	“[21]nstability*”[Title/Abstract] OR “dislocation*”[Title/Abstract] OR “subluxation*”[Title/Abstract] OR “luxation*”[Title/Abstract]	214,439
4	(“Joint Instability”[MeSH Terms] OR “Athletic Injuries”[MeSH Terms]) AND (“shoulder”[Title/Abstract] OR “glenohum*”[Title/Abstract] OR “gleno hum*”[Title/Abstract] OR “labrum”[Title/Abstract])	6236
3	“shoulder”[Title/Abstract] OR “glenohum*”[Title/Abstract] OR “gleno hum*”[Title/Abstract] OR “labrum”[Title/Abstract]	8763
2	“Joint Instability”[MeSH Terms] OR “Athletic Injuries”[MeSH Terms]	53,531
1	“Shoulder Dislocation”[MeSH Terms] OR “Bankart Lesions”[MeSH Terms]	6708

**Embase:**

**No.**	**Query**	**Results**
#24	(((‘shoulder dislocation’/exp OR ‘bankart lesion’/exp) OR ((‘joint instability’/de OR ‘sport injury’/exp) AND (shoulder:ti,ab,kw OR glenohum*:ti,ab,kw OR ‘gleno hum*’:ti,ab,kw OR labrum:ti,ab,kw)) OR ((shoulder:ti,ab,kw OR glenohum*:ti,ab,kw OR ‘gleno hum*’:ti,ab,kw OR labrum:ti,ab,kw) AND ([21]nstability*:ti,ab,kw OR dislocation*:ti,ab,kw OR subluxation*:ti,ab,kw OR luxation*:ti,ab,kw)) OR (‘bankart lesion*’:ti,ab,kw OR ‘bankart fracture*’:ti,ab,kw)) AND anterior:ti,ab,kw AND ((‘orthopedics’/exp OR ‘orthopedic surgery’/exp OR ‘conservative treatment’/exp) OR (operat*:ti,ab,kw OR surger*:ti,ab,kw OR surgic*:ti,ab,kw OR ‘bankart repair*’:ti,ab,kw OR ‘bankart procedure*’:ti,ab,kw OR ‘latarjet-bristow*’:ti,ab,kw OR conservative:ti,ab,kw OR immobilis*:ti,ab,kw OR immobiliz*:ti,ab,kw OR ‘collar and cuff’:ti,ab,kw OR ‘collarandcuff’:ti,ab,kw OR ‘non surg*’:ti,ab,kw OR nonsurg*:ti,ab,kw OR ‘non operat*’:ti,ab,kw OR nonoperat*:ti,ab,kw)) AND ((‘athlete’/exp OR ‘sport’/exp OR ‘sport injury’/exp) OR (athlete*:ti,ab,kw OR sport*:ti,ab,kw OR ‘competition*’:ti,ab,kw OR play:ti,ab,kw OR collision*:ti,ab,kw)) NOT ([conference abstract]/lim OR [preprint]/lim) AND ([[21]nstab]/lim OR [[21]nstabi]/lim) AND [2013–2023]/py) AND ((‘treatment outcome’/exp OR ‘return to play’/exp OR ‘return to sport’/exp) OR (‘return to play*’:ti,ab,kw OR ‘return to sport*’:ti,ab,kw OR ‘return to competition*’:ti,ab,kw OR outcome:ti,ab,kw OR resumption*:ti,ab,kw OR ‘treatment efficacy’:ti,ab,kw))	506
#23	(‘treatment outcome’/exp OR ‘return to play’/exp OR ‘return to sport’/exp) OR (‘return to play*’:ti,ab,kw OR ‘return to sport*’:ti,ab,kw OR ‘return to competition*’:ti,ab,kw OR outcome:ti,ab,kw OR resumption*:ti,ab,kw OR ‘treatment efficacy’:ti,ab,kw)	3,532,572
#22	‘return to play*’:ti,ab,kw OR ‘return to sport*’:ti,ab,kw OR ‘return to competition*’:ti,ab,kw OR outcome:ti,ab,kw OR resumption*:ti,ab,kw OR ‘treatment efficacy’:ti,ab,kw	1,943,508
#21	‘treatment outcome’/exp OR ‘return to play’/exp OR ‘return to sport’/exp	2,328,958
#20	((‘shoulder dislocation’/exp OR ‘bankart lesion’/exp) OR ((‘joint instability’/de OR ‘sport injury’/exp) AND (shoulder:ti,ab,kw OR glenohum*:ti,ab,kw OR ‘gleno hum*’:ti,ab,kw OR labrum:ti,ab,kw)) OR ((shoulder:ti,ab,kw OR glenohum*:ti,ab,kw OR ‘gleno hum*’:ti,ab,kw OR labrum:ti,ab,kw) AND ([22]nstability*:ti,ab,kw OR dislocation*:ti,ab,kw OR subluxation*:ti,ab,kw OR luxation*:ti,ab,kw)) OR (‘bankart lesion*’:ti,ab,kw OR ‘bankart fracture*’:ti,ab,kw)) AND anterior:ti,ab,kw AND ((‘orthopedics’/exp OR ‘orthopedic surgery’/exp OR ‘conservative treatment’/exp) OR (operat*:ti,ab,kw OR surger*:ti,ab,kw OR surgic*:ti,ab,kw OR ‘bankart repair*’:ti,ab,kw OR ‘bankart procedure*’:ti,ab,kw OR ‘latarjet-bristow*’:ti,ab,kw OR conservative:ti,ab,kw OR immobilis*:ti,ab,kw OR immobiliz*:ti,ab,kw OR ‘collar and cuff’:ti,ab,kw OR ‘collarandcuff’:ti,ab,kw OR ‘non surg*’:ti,ab,kw OR nonsurg*:ti,ab,kw OR ‘non operat*’:ti,ab,kw OR nonoperat*:ti,ab,kw)) AND ((‘athlete’/exp OR ‘sport’/exp OR ‘sport injury’/exp) OR (athlete*:ti,ab,kw OR sport*:ti,ab,kw OR ‘competition*’:ti,ab,kw OR play:ti,ab,kw OR collision*:ti,ab,kw)) NOT ([conference abstract]/lim OR [preprint]/lim) AND ([[22]nstab]/lim OR [[22]nstabi]/lim) AND [2013–2023]/py	Uden den sidste blok “Outcome” block left out 724
#19	((‘shoulder dislocation’/exp OR ‘bankart lesion’/exp) OR ((‘joint instability’/de OR ‘sport injury’/exp) AND (shoulder:ti,ab,kw OR glenohum*:ti,ab,kw OR ‘gleno hum*’:ti,ab,kw OR labrum:ti,ab,kw)) OR ((shoulder:ti,ab,kw OR glenohum*:ti,ab,kw OR ‘gleno hum*’:ti,ab,kw OR labrum:ti,ab,kw) AND ([22]nstability*:ti,ab,kw OR dislocation*:ti,ab,kw OR subluxation*:ti,ab,kw OR luxation*:ti,ab,kw)) OR (‘bankart lesion*’:ti,ab,kw OR ‘bankart fracture*’:ti,ab,kw)) AND anterior:ti,ab,kw AND ((‘orthopedics’/exp OR ‘orthopedic surgery’/exp OR ‘conservative treatment’/exp) OR (operat*:ti,ab,kw OR surger*:ti,ab,kw OR surgic*:ti,ab,kw OR ‘bankart repair*’:ti,ab,kw OR ‘bankart procedure*’:ti,ab,kw OR ‘latarjet-bristow*’:ti,ab,kw OR conservative:ti,ab,kw OR immobilis*:ti,ab,kw OR immobiliz*:ti,ab,kw OR ‘collar and cuff’:ti,ab,kw OR ‘collarandcuff’:ti,ab,kw OR ‘non surg*’:ti,ab,kw OR nonsurg*:ti,ab,kw OR ‘non operat*’:ti,ab,kw OR nonoperat*:ti,ab,kw)) AND ((‘athlete’/exp OR ‘sport’/exp OR ‘sport injury’/exp) OR (athlete*:ti,ab,kw OR sport*:ti,ab,kw OR ‘competition*’:ti,ab,kw OR play:ti,ab,kw OR collision*:ti,ab,kw)) NOT ([conference abstract]/lim OR [preprint]/lim) AND ([[22]nstab]/lim OR [[22]nstabi]/lim)	1171
#18	((‘shoulder dislocation’/exp OR ‘bankart lesion’/exp) OR ((‘joint instability’/de OR ‘sport injury’/exp) AND (shoulder:ti,ab,kw OR glenohum*:ti,ab,kw OR ‘gleno hum*’:ti,ab,kw OR labrum:ti,ab,kw)) OR ((shoulder:ti,ab,kw OR glenohum*:ti,ab,kw OR ‘gleno hum*’:ti,ab,kw OR labrum:ti,ab,kw) AND ([23]nstability*:ti,ab,kw OR dislocation*:ti,ab,kw OR subluxation*:ti,ab,kw OR luxation*:ti,ab,kw)) OR (‘bankart lesion*’:ti,ab,kw OR ‘bankart fracture*’:ti,ab,kw)) AND anterior:ti,ab,kw AND ((‘orthopedics’/exp OR ‘orthopedic surgery’/exp OR ‘conservative treatment’/exp) OR (operat*:ti,ab,kw OR surger*:ti,ab,kw OR surgic*:ti,ab,kw OR ‘bankart repair*’:ti,ab,kw OR ‘bankart procedure*’:ti,ab,kw OR ‘latarjet-bristow*’:ti,ab,kw OR conservative:ti,ab,kw OR immobilis*:ti,ab,kw OR immobiliz*:ti,ab,kw OR ‘collar and cuff’:ti,ab,kw OR ‘collarandcuff’:ti,ab,kw OR ‘non surg*’:ti,ab,kw OR nonsurg*:ti,ab,kw OR ‘non operat*’:ti,ab,kw OR nonoperat*:ti,ab,kw)) AND ((‘athlete’/exp OR ‘sport’/exp OR ‘sport injury’/exp) OR (athlete*:ti,ab,kw OR sport*:ti,ab,kw OR ‘competition*’:ti,ab,kw OR play:ti,ab,kw OR collision*:ti,ab,kw)) NOT ([conference abstract]/lim OR [preprint]/lim)	1272
#17	((‘shoulder dislocation’/exp OR ‘bankart lesion’/exp) OR ((‘joint instability’/de OR ‘sport injury’/exp) AND (shoulder:ti,ab,kw OR glenohum*:ti,ab,kw OR ‘gleno hum*’:ti,ab,kw OR labrum:ti,ab,kw)) OR ((shoulder:ti,ab,kw OR glenohum*:ti,ab,kw OR ‘gleno hum*’:ti,ab,kw OR labrum:ti,ab,kw) AND ([23]nstability*:ti,ab,kw OR dislocation*:ti,ab,kw OR subluxation*:ti,ab,kw OR luxation*:ti,ab,kw)) OR (‘bankart lesion*’:ti,ab,kw OR ‘bankart fracture*’:ti,ab,kw)) AND anterior:ti,ab,kw AND ((‘orthopedics’/exp OR ‘orthopedic surgery’/exp OR ‘conservative treatment’/exp) OR (operat*:ti,ab,kw OR surger*:ti,ab,kw OR surgic*:ti,ab,kw OR ‘bankart repair*’:ti,ab,kw OR ‘bankart procedure*’:ti,ab,kw OR ‘latarjet-bristow*’:ti,ab,kw OR conservative:ti,ab,kw OR immobilis*:ti,ab,kw OR immobiliz*:ti,ab,kw OR ‘collar and cuff’:ti,ab,kw OR ‘collarandcuff’:ti,ab,kw OR ‘non surg*’:ti,ab,kw OR nonsurg*:ti,ab,kw OR ‘non operat*’:ti,ab,kw OR nonoperat*:ti,ab,kw)) AND ((‘athlete’/exp OR ‘sport’/exp OR ‘sport injury’/exp) OR (athlete*:ti,ab,kw OR sport*:ti,ab,kw OR ‘competition*’:ti,ab,kw OR play:ti,ab,kw OR collision*:ti,ab,kw)) AND ([conference abstract]/lim OR [preprint]/lim)	234
#16	((‘shoulder dislocation’/exp OR ‘bankart lesion’/exp) OR ((‘joint instability’/de OR ‘sport injury’/exp) AND (shoulder:ti,ab,kw OR glenohum*:ti,ab,kw OR ‘gleno hum*’:ti,ab,kw OR labrum:ti,ab,kw)) OR ((shoulder:ti,ab,kw OR glenohum*:ti,ab,kw OR ‘gleno hum*’:ti,ab,kw OR labrum:ti,ab,kw) AND ([23]nstability*:ti,ab,kw OR dislocation*:ti,ab,kw OR subluxation*:ti,ab,kw OR luxation*:ti,ab,kw)) OR (‘bankart lesion*’:ti,ab,kw OR ‘bankart fracture*’:ti,ab,kw)) AND anterior:ti,ab,kw AND ((‘orthopedics’/exp OR ‘orthopedic surgery’/exp OR ‘conservative treatment’/exp) OR (operat*:ti,ab,kw OR surger*:ti,ab,kw OR surgic*:ti,ab,kw OR ‘bankart repair*’:ti,ab,kw OR ‘bankart procedure*’:ti,ab,kw OR ‘latarjet-bristow*’:ti,ab,kw OR conservative:ti,ab,kw OR immobilis*:ti,ab,kw OR immobiliz*:ti,ab,kw OR ‘collar and cuff’:ti,ab,kw OR ‘collarandcuff’:ti,ab,kw OR ‘non surg*’:ti,ab,kw OR nonsurg*:ti,ab,kw OR ‘non operat*’:ti,ab,kw OR nonoperat*:ti,ab,kw)) AND ((‘athlete’/exp OR ‘sport’/exp OR ‘sport injury’/exp) OR (athlete*:ti,ab,kw OR sport*:ti,ab,kw OR ‘competition*’:ti,ab,kw OR play:ti,ab,kw OR collision*:ti,ab,kw))	1506
#15	(‘athlete’/exp OR ‘sport’/exp OR ‘sport injury’/exp) OR (athlete*:ti,ab,kw OR sport*:ti,ab,kw OR ‘competition*’:ti,ab,kw OR play:ti,ab,kw OR collision*:ti,ab,kw)	1,500,389
#14	athlete*:ti,ab,kw OR sport*:ti,ab,kw OR ‘competition*’:ti,ab,kw OR play:ti,ab,kw OR collision*:ti,ab,kw	1,353,407
#13	‘athlete’/exp OR ‘sport’/exp OR ‘sport injury’/exp	278,953
#12	(‘orthopedics’/exp OR ‘orthopedic surgery’/exp OR ‘conservative treatment’/exp) OR (operat*:ti,ab,kw OR surger*:ti,ab,kw OR surgic*:ti,ab,kw OR ‘bankart repair*’:ti,ab,kw OR ‘bankart procedure*’:ti,ab,kw OR ‘latarjet-bristow*’:ti,ab,kw OR conservative:ti,ab,kw OR immobilis*:ti,ab,kw OR immobiliz*:ti,ab,kw OR ‘collar and cuff’:ti,ab,kw OR ‘collarandcuff’:ti,ab,kw OR ‘non surg*’:ti,ab,kw OR nonsurg*:ti,ab,kw OR ‘non operat*’:ti,ab,kw OR nonoperat*:ti,ab,kw)	5,350,881
#11	operat*:ti,ab,kw OR surger*:ti,ab,kw OR surgic*:ti,ab,kw OR ‘bankart repair*’:ti,ab,kw OR ‘bankart procedure*’:ti,ab,kw OR ‘latarjet-bristow*’:ti,ab,kw OR conservative:ti,ab,kw OR immobilis*:ti,ab,kw OR immobiliz*:ti,ab,kw OR ‘collar and cuff’:ti,ab,kw OR ‘collarandcuff’:ti,ab,kw OR ‘non surg*’:ti,ab,kw OR nonsurg*:ti,ab,kw OR ‘non operat*’:ti,ab,kw OR nonoperat*:ti,ab,kw	4,505,702
#10	‘orthopedics’/exp OR ‘orthopedic surgery’/exp OR ‘conservative treatment’/exp	1,338,529
#9	anterior:ti,ab,kw	568,396
#8	(’shoulder dislocation’/exp OR ’bankart lesion’/exp) OR ((’joint instability’/de OR ’sport injury’/exp) AND (shoulder:ti,ab,kw OR glenohum*:ti,ab,kw OR ’gleno hum*’:ti,ab,kw OR labrum:ti,ab,kw)) OR ((shoulder:ti,ab,kw OR glenohum*:ti,ab,kw OR ’gleno hum*’:ti,ab,kw OR labrum:ti,ab,kw) AND (instabilit*:ti,ab,kw OR dislocation*:ti,ab,kw OR subluxation*:ti,ab,kw OR luxation*:ti,ab,kw)) OR (’bankart lesion*’:ti,ab,kw OR ’bankart fracture*’:ti,ab,kw)	20,395
#7	’bankart lesion*’:ti,ab,kw OR ’bankart fracture*’:ti,ab,kw	964
#6	(shoulder:ti,ab,kw OR glenohum*:ti,ab,kw OR ’gleno hum*’:ti,ab,kw OR labrum:ti,ab,kw) AND (instabilit*:ti,ab,kw OR dislocation*:ti,ab,kw OR subluxation*:ti,ab,kw OR luxation*:ti,ab,kw)	15,787
#5	instabilit*:ti,ab,kw OR dislocation*:ti,ab,kw OR subluxation*:ti,ab,kw OR luxation*:ti,ab,kw	259,475
#4	(’joint instability’/de OR ’sport injury’/exp) AND (shoulder:ti,ab,kw OR glenohum*:ti,ab,kw OR ’gleno hum*’:ti,ab,kw OR labrum:ti,ab,kw)	4770
#3	shoulder:ti,ab,kw OR glenohum*:ti,ab,kw OR ’gleno hum*’:ti,ab,kw OR labrum:ti,ab,kw	110,037
#2	‘joint instability’/de OR ‘sport injury’/exp	50,611
#1	‘shoulder dislocation’/exp OR ‘bankart lesion’/exp	8414

**Cochrane Library:**

**ID**	**Search**	**Hits**
#1	MeSH descriptor: [Shoulder Dislocation] explode all trees	213
#2	MeSH descriptor: [Bankart Lesions] explode all trees	14
#3	MeSH descriptor: [Joint Instability] explode all trees	983
#4	MeSH descriptor: [Athletic Injuries] explode all trees	902
#5	#3 OR #4	1849
#6	(shoulder OR glenohum* OR gleno-hum* OR labrum):ti,ab,kw	15,967
#7	#5 AND #6	176
#8	(Bankart NEXT lesion*):ti,ab,kw	71
#9	(Bankart NEXT fracture*):ti,ab,kw	2
#10	#1 OR #2 OR #7 OR #8 OR #9	389
#11	(Anterior):ti,ab,kw	34,315
#12	MeSH descriptor: [Orthopedics] explode all trees	556
#13	MeSH descriptor: [Surgical Procedures, Operative] explode all trees	166,595
#14	MeSH descriptor: [Conservative Treatment] explode all trees	650
#15	(operat* OR surger* OR surgic* OR (Bankart NEXT repair*) OR (Bankart NEXT procedure*) OR Latarjet-Bristow* OR conservative OR immobilis* OR immobiliz* OR “collar and cuff” OR collarandcuff OR non-surg* OR nonsurg* OR non-operat* OR nonoperat*):ti,ab,kw	362,496
#16	#12 OR #13 OR #14 OR #15	418,111
#17	MeSH descriptor: [Athletes] explode all trees	1483
#18	MeSH descriptor: [Sports] explode all trees	21,015
#19	MeSH descriptor: [Athletic Injuries] explode all trees	902
#20	(athlete* OR sport* OR competition* OR play OR collision*):ti,ab,kw	40,971
#21	#17 OR #18 OR #19 OR #20	56,847
#22	MeSH descriptor: [Treatment Outcome] explode all trees	182,091
#23	MeSH descriptor: [Return to Sport] explode all trees	73
#24	(“return to play” OR “return to sport” OR “return to competition” OR outcome OR resumption* OR “treatment efficacy”):ti,ab,kw	668,098
#25	#22 OR #23 OR #24	675,791
#26	#10 AND #11 AND #16 AND #21	37 “Outcome” -block left out
#27	#26 AND #25	27 (2 reviews 25 trials)
